# The Association of COPD Exacerbations with New Onset Type 2 Diabetes among Medicare Patients

**DOI:** 10.36469/9810

**Published:** 2017-11-29

**Authors:** Joseph S. Marino, Cynthiya Ruban, Christopher M. Blanchette

**Affiliations:** 1 Health Informatics and Outcomes Research Academy, Laboratory of Systems Physiology, Department of Kinesiology University of North Carolina, Charlotte, NC, USA; 2 Health Informatics and Outcomes Research Academy, Department of Public Health Sciences University of North Carolina, Charlotte, NC, USA

**Keywords:** drug safety, chronic obstructive pulmonary disease, corticosteroid, medicare, type 2 diabetes

## Abstract

**Objective:** Chronic obstructive pulmonary disease (COPD) is highly prevalent in the elderly population and typically reduces overall quality of life. Exacerbations of COPD are commonly treated with corticosteroids, a class of drug known to cause insulin resistance. The objective of this study was to assess the rate of exacerbations requiring emergency room visits, hospitalizations or any medical encounter (a combination of emergency room and hospitalizations) between COPD patients who did and did not develop type 2 diabetes.

**Research Design and Methods:** A case-control study of COPD patients from the 2011-2012 Medicare 5% sample Limited Data Set (LDS) was conducted. Beneficiaries with at least 1 year of continuous enrollment and evidence of > 2 COPD-related claims (>1 primary diagnosis) were included in the study. Cases were defined as a beneficiary with a new claim for type 2 diabetes, whereas controls lacked evidence of type 2 diabetes (beneficiaries with evidence of non-incident type 2 diabetes were excluded).

**Results:** Of 27 456 COPD beneficiaries, 1274 developed incident type 2 diabetes (4.6%). After matching, 2536 beneficiaries were assigned as cases (n = 1268) and controls (n = 1268). Cases in the emergency room (1.97 claims per person) (p = <0.001) and hospitalizations (2.02 claims per person) (p = <0.001) had a higher rate of exacerbations.

**Conclusion:** Our findings suggest that patients that were hospitalized and visited the emergency room for COPD exacerbations had a greater likelihood of type 2 diabetes. Type 2 diabetes may be associated with exposure to corticosteroids as a result of the treatment for exacerbations. Future work should investigate the risk for type 2 diabetes in COPD patients treated with corticosteroids.

## INTRODUCTION

Chronic Obstructive Pulmonary Disease (COPD) affects 24 million Americans and causes a substantial reduction in quality of life and independence due to difficulty breathing, low hemoglobin saturation, and exacerbation-induced hospitalization. COPD exacerbations are commonly treated with glucocorticoids, which are steroid therapies that fall into the corticosteroid family. While glucocorticoid therapy is effective in managing COPD symptoms, and are routinely used to treat exacerbations, their actions are not specific to COPD pathophysiology. Therefore, systemic glucocorticoid treatments may alter the activity of glucocorticoid receptors in other tissues, resulting in physiological responses that are not favorable in tissues other than the respiratory tract. Skeletal muscle, the largest metabolically active tissue in the body, is a primary example of a tissue that is adversely affected by chronic glucocorticoid exposure resulting from COPD treatment.[1]

It is known that chronic skeletal muscle exposure to glucocorticoid adversely affects the canonical insulin signaling pathway. This pathway regulates the balance between protein synthesis and degradation, which could impact skeletal muscle repair, maintenance of size, and function.[2-5] In addition, the insulin signaling pathway is the primary regulator of post-prandial glucose disposal, such that skeletal muscle insulin resistance reduces glucose transport from the blood, resulting in hyperglycemia.[6] Glucocorticoid-mediated modulation of metabolic function in skeletal muscle requires the interaction between glucocorticoid and the glucocorticoid receptor, leading to nuclear localization and docking on glucocorticoid response elements (GREs) that regulate gene activity.[1,7,8] Importantly, skeletal muscle cells have several GREs, many of which directly impair insulin sensitivity.[7-9]

These studies highlight insulin resistance and type 2 diabetes (T2D) as potential devastating side effects of glucocorticoid-based treatments for COPD. Approximately 10% of COPD patients will develop T2D.[10] COPD is associated with heightened complexity of care in those with diabetes, a potential result of decreased ability to perform self-management activities necessary for optimal diabetes control and the associated therapeutic challenges.[11] However, the prevalence of the development of T2D and dose response relationship in those undergoing treatment for COPD with corticosteroids is not well established. Present treatment recommendations for COPD include the usage of inhaled corticosteroids to reduce exacerbations in patients with COPD that is moderate to severe.[11] Oral corticosteroids are also suggested for the short-term treatment of exacerbations to enhance lung function and hypoxemia and to decrease recovery time but are not recommended chronically due to their unfavorable risk/benefits profile.[11] Corticosteroids are not advised for patients with diabetes[12] because they cause dose-dependent increases in blood glucose levels, enhancing the risk of diabetes progression.[13]

The objective of this study was to assess the rate of exacerbations requiring emergency room visits, hospitalizations, or any medical encounter (a combination of emergency room and hospitalizations) between COPD patients who did and did not develop type 2 diabetes.

## RESEARCH DESIGNS AND METHODS

### Data sources

COPD patients in the 2011-2012 Medicare 5% sample Limited Data Set (LDS) were observed. Medicare beneficiaries represent 16% of the total US population or approximately 51 million individuals covered under Part A (hospital) and Part B (outpatient services). Medicare data is available in a range of formats with the most usual being the LDS 5% sample and the Research Identifiable Files (RIF). The LDS includes a set random sample of 5% of the Medicare Fee-for-Service population, which can be stacked year over year as a standard extract. The RIF are custom extracts and can be extracted up to a 100% sample, provided justification for the request is approved by the Centers for Medicare and Medicaid Services (CMS).

### Cohort selection

A case-control study of COPD patients from the 2011-2012 Medicare 5% sample LDS was conducted. Beneficiaries with at least 1 year of continuous enrollment and evidence of ≥ 2 COPD-related claims (≥1 primary diagnosis) were included in the study. COPD was defined by ICD-9-CM codes 491, 492, 496. These included chronic bronchitis, emphysema, and chronic airway obstruction. Cases were defined as a beneficiary with incident type 2 diabetes, whereas controls lacked evidence of type 2 diabetes (beneficiaries with evidence of non-incident type 2 diabetes were excluded). Cases and controls were matched on their propensity to have a new claim for type 2 diabetes using the Greedy matching algorithm.[14] The Greedy matching algorithm was employed to adjust for preindex heterogeneity in observed patients' characteristics and health care utilization rates.

### Outcome definition

The study end point was rate of claims (claim per person), with a primary diagnosis of COPD, from the index COPD date to the index diabetes date. Diabetes was defined by the ICD-9-CM codes 250.00 and 250.02. These include diabetes mellitus without mention of complication, type II or unspecified type, and uncontrolled. Follow-up commenced 12 months from index of COPD diagnosis. Participants were censored at death or end of observation. The rate (claims per person) of moderate (emergency department visits) and severe (hospitalizations) exacerbations, with COPD as a primary diagnosis, was utilized as a proxy measure for corticosteroid exposure.

### Statistical Analysis

All analyses were performed using SAS® (version 9.4) software. Propensity scores were used to estimate risk for diabetes in each patient based on patient demographics and preexisting and newly diagnosed chronic medical conditions other than diabetes and COPD. Pre-existing and newly diagnosed chronic medical conditions were defined using Elixhauser comorbidity measure. The Elixhauser comorbidity measure provides a dichotomous comorbidity flag utilizing International Classification of Diseases (ICD-9) codes.[15] The propensity score can be defined as the probability of being in the case group given the individual's level on the covariates included in the model.[16] The propensity score is frequently estimated using a logistic regression model as logistic regression does not infer assumptions regarding the distributions of the covariates on the dichotomous outcome.[17] The variables were selected for matching patients who developed diabetes (cases) with those who did not (controls) at a 1:1 ratio. This was done to secure a well-balanced analysis set necessary to study the effect of our exposure of interest (steroid use) on our outcome of interest (development of incident diabetes).

Propensity score matching uses the structure of matching two groups to make them analogous, but pairs them on one indicator - the propensity score - instead of multiple variables. When matching, controls from the non-diabetes group are selected who have similar propensity scores to those in the cases. This propensity score is then utilized to adjust for the variances among both groups on the observed covariates in the study. Thus, the propensity score is often a means of balancing a score allowing researchers to control for a vast number of covariates simultaneously based on a single number.[16]

For the next part of the analysis, the two groups of matched patients were compared on the outcome variables of primary interest. Estimates of patients' propensity scores were obtained using logistic regression. For matching patients on the propensity scores, one-to-one nearest neighbor matching was used. Once matched, the average number of primary COPD claims were compared with their matched counterparts who did not develop diabetes, using paired samples t-tests. Descriptive and bivariate statistics were used to describe baseline patient characteristics of comparator cohorts pre- and post-matching. Bivariate statistics were used to assess association between development of incident diabetes and steroid exposure (via the three different types of exacerbations).

## RESULTS

After removing patients with diabetes in the pre-period, 15 117 COPD beneficiaries were initially identified. Of those, 1274 beneficiaries developed T2D. Prior to matching both groups, patients without T2D were statistically older (73.04±11.98) compared to patients with T2D (71.3±11.2) ($P < 0.001$). There were also statistical differences in gender and race ($P < 0.001$) between both groups. Differences in comorbidities between the groups included significantly higher congestive heart failure, arrhythmias, renal failure and obesity ($P < 0.001$) (Table 1).

After matching, 2546 beneficiaries were assigned to cases ($n = 1273$) and controls ($n = 1273$). The propensity scores ranged from 0.0595 to 0.4440 across the sample. Cases and controls were well balanced with respect to all of the measured covariates (Table 2).

Rate of exacerbations was assessed in three settings: emergency room, hospitalizations, and any setting (a combination of emergency room and hospitalizations.) Cases had higher rate of any exacerbations in the emergency room (1.97 claims per person) ($p = <0.001$) (OR =1.103) and hospitalizations (2.02 claims per person) ($p = <0.001$) (OR=1.64). There was a statistically higher number of hospitalizations amongst the COPD-T2D population, compared to the COPD population, in three different settings (Table 3). Histograms of the distributions of cases and controls are presented in Figure 1.

**Table 1.** Baseline Characteristics Prior to Matching

| PATIENT DEMOGRAPHICS | No Diabetes N=11 466 | Diabetes N=1274 | SIGNIFICANCE |
|---|---|---|---|
| Age (mean±SD) | 73.04 ±11.98 | 71.3±11.2 | *P* < 0.001 |
| Gender (n, %) | - | - | *P* < 0.001 |
| Male | 5,362 (46.8%) | 708 (55.6%) | - |
| Female | 6,104 (53.2%) | 566 (44.4%) | - |
| Race (n, %) | - | - | *P* < 0.001 |
| White | 10,211 (89.0%) | 1,037 (81.4%) | - |
| Black | 869 (7.6%) | 171 (13.4%) | - |
| Other | 70 (0.6%) | 10 (0.8%) | - |
| Asian | 109(1.0%) | 14 (1.1%) | - |
| Hispanic | 140 (1.2%) | 26 (2.0%) | - |
| NA Native | 67 (0.6%) | 16 (1.3%) | - |
| Elixhauser | | | |
| Congestive heart failure | 579 (5.0%) | 130 (10.2%) | *P* <.0001 |
| Arrythmias | 3074 (26.8%) | 439 (34.5%) | *P* <.0001 |
| Valve disorder | 259 (2.3%) | 25 (2.0%) | 0.7195 |
| Pulmonary circulation disorder | 208 (1.8%) | 23 (1.8%) | 0.6443 |
| Peri-Vascular disorder | 877 (7.7%) | 147 (11.5%) | *P* <.0001 |
| Paralysis | 39 (0.3%) | 6 (0.5%) | 0.9467 |
| Other neurological disorders | 783 (6.8%) | 112 (8.8%) | 0.0031 |
| Hypothyroidism | 2 (0.0%) | 8 (0.6%) | 0.2599 |
| Renal failure | 194 (1.7%) | 60 (4.7%) | *P* <.0001 |
| Liver disease | 376 (3.3%) | 48 (3.8%) | 0.0126 |
| Peptic ulcer no bleed | 199 (1.7%) | 29 (2.3%) | 0.7358 |
| HIV/AIDS | 40 (0.3%) | 5 (0.4%) | 0.031 |
| Nonmetastatic tumor | 235 (2.0%) | 22 (1.7%) | 0.9989 |
| Rheumatoid arthritis | 195 (1.7%) | 13 (1.0%) | 0.9185 |
| Coagulopathy | 446 (3.9%) | 73 (5.7%) | 0.0014 |
| Obesity | 910 (7.9%) | 246 (19.3%) | *P* <.0001 |
| Weightloss | 605 (5.3%) | 75 (5.9%) | 0.0059 |
| Fluid and electrolyte disorders | 76 (0.7%) | 4 (0.3%) | 0.136 |
| Blood loss anemia | 491 (4.3%) | 73 (5.7%) | 0.0586 |
| Deficiency anemia | 491 (4.3%) | 16 (1.3%) | 0.0209 |
| Alcohol abuse | 571 (5.0%) | 83 (6.5%) | 0.0611 |
| Drug abuse | 224 (1.9%) | 71 (5.6%) | 0.0424 |
| Psychoses | 25 (0.2%) | 29 (2.3%) | 0.2147 |
| Depression | 1774(15.5%) | 239 (18.8%) | 0.0017 |

SD: standard deviation; NA: North American; HIV/AIDS: human immunovirus/acquired immune deficiency syndrome

**Table 2.** Baseline Characteristics after Matching

| PATIENT DEMOGRAPHICS | No Diabetes N=1273 | Diabetes N=1273 | SIGNIFICANCE |
|---|---|---|---|
| Age (mean±SD) | 72.04±11.10 | 71.31±11.22 | *P* = 0.1014 |
| Gender (n, %) | - | - | |
| Male | 739 (58.1%) | 708 (55.6%) | *P* = 0.2148 |
| Female | 534 (41.9%) | 565 (44.4%) | |
| Race (n, %) | - | - | *P* = 0.3972 |
| White | 1028 (80.8%) | 1036 (81.4%) | - |
| Black | 172 (13.5%) | 171 (13.4%) | - |
| Other | 13 (1.0%) | 10 (0.8%) | - |
| Asian | 22 (1.7%) | 14 (1.1%) | - |
| Hispanic | 30 (2.4%) | 26 (2.0%) | - |
| NA Native | 8 (0.6%) | 16 (1.3%) | - |
| Elixhauser | | | |
| Congestive heart failure | 84 (6.6%) | 130 (10.2%) | 0.1025 |
| Arrhythmias | 935 (73.4%) | 439 (34.4%) | 0.039 |
| Valve disorder | 32 (2.5%) | 25 (2.0%) | 0.4454 |
| Pulmonary circulation disorder | 25 (2.0%) | 22 (1.7%) | 0.6074 |
| Peri-Vascular disorder | 156 (12.3%) | 147 (11.5%) | 0.708 |
| Paralysis | 7 (0.5%) | 6 (0.5%) | 0.4529 |
| Other neurological disorders | 67 (5.3%) | 112 (8.8%) | 0.0044 |
| Hypothyroidism | 9 (0.7%) | 7 (0.5%) | 0.6106 |
| Renal failure | 33 (2.6%) | 60 (4.7%) | 0.0156 |
| Liver disease | 38 (3.0%) | 48 (3.8%) | 0.4492 |
| Peptic ulcer no bleed | 20 (1.6%) | 29 (2.3%) | 0.3208 |
| HIV/AIDS | 4 (0.3%) | 5 (0.4%) | 0.3208 |
| Nonmetastatic tumor | 38 (3.0%) | 22 (1.7%) | 0.3041 |
| Rheumatoid arthritis | 21 (1.6%) | 13 (1.0%) | 0.3484 |
| Coagulopathy | 49 (3.8%) | 73 (5.7%) | 0.2447 |
| Obesity | 214 (16.8%) | 245 (19.2%) | 0.4136 |
| Weightloss | 68 (5.3%) | 75 (5.9%) | 0.6862 |
| Fluid and electrolyte disorders | 12 (0.9%) | 4 (0.3%) | 0.0261 |
| Blood loss anemia | 21 (1.6%) | 16 (1.3%) | 0.5089 |
| Deficiency anemia | 62 (4.9%) | 83 (6.5%) | 0.2979 |
| Alcohol abuse | 61 (4.8%) | 71 (5.6%) | 0.4193 |
| Drug abuse | 27 (2.1%) | 29 (2.3%) | 0.4681 |
| Psychoses | 1 (0.1%) | 6 (0.5%) | 0.1591 |
| Depression | 202 (16%) | 238 (18.7%) | 0.2149 |

SD: standard deviation; NA: North American; HIV/AIDS: human immunovirus/acquired immune deficiency syndrome

**Figure 1.** Histograms of Exacerbations
The frequency of hospitalization and emergency room care for A) non-diabetics and B) those that developed diabetes. Statistical analysis of differences between groups is presented in Table 3.

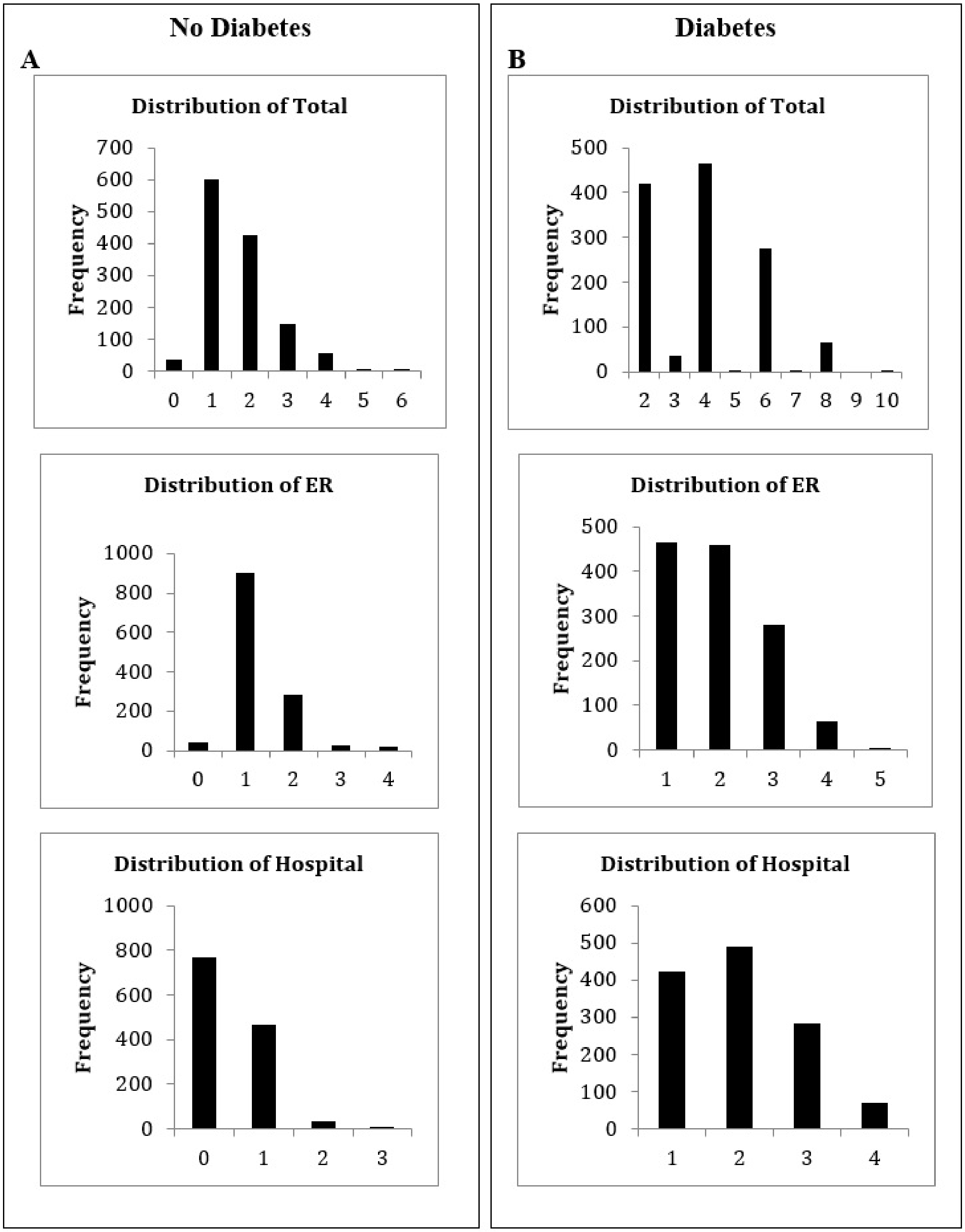

ER: emergency room

**Table 3.** Rate of Exacerbations

| | No Diabetes (Claim per person per year) | Diabetes (Claim per person per year) | Significance |
|---|---|---|---|
| | **N=1273** | **N=1273** | |
| Emergency Room | 1.272 | 1.970 | $P < 0.001$ |
| Hospitalizations | 0.412 | 2.015 | $P < 0.001$ |
| Any Setting | 1.680 | 3.988 | $P < 0.001$ |

## CONCLUSION

Our findings suggest that patients that were hospitalized or visited any medical setting for COPD had increased type 2 diabetes claims.

Type 2 diabetes and COPD are prevalent comorbid disorders, and both the prevalence and incidence of type 2 diabetes are higher in patients with COPD, in relation with those without.[18,19] The occurrence of both type 2 diabetes and COPD has been reported to be associated with elevated care complexity.[11] Elevated numbers of comorbid conditions or the presence of comorbid cardiovascular diseases, such as hypertension, depression, or anxiety are correlated with increased respiratory impairment and risk of hospitalization and mortality.[19,20] Our results highlight the significance of shared decision making in this specific population, where it is essential for treatment prioritization of the most symptomatic conditions, such as COPD, to be coordinated with both the short and long-term harm associated with usage of corticosteroids for patients with comorbid diabetes.

Previous studies have demonstrated that corticosteroids are associated with insulin resistance, loss of diabetic control in a dose-dependent manner, and hyperglycemia.[13,21] Blackburn et al. conducted a population based cohort study utilizing an administrative database. Findings from this study documented that oral corticosteroid users were significantly more likely than Proton pump inhibitor users to develop diabetes mellitus (adjusted rate ratio, 2.31; 95% CI 2.11 to 2.54). However, the risk of inhaled corticosteroid users was not significantly different from that of Proton pump inhibitor users. Findings support the association between the use of oral corticosteroids and the development of diabetes mellitus.

Corticosteroids have demonstrated inhibition of a number of steps in the insulin signaling network which can lead to insulin resistance. These include increased lipolysis, proteolysis, and free fatty acid production. Moreover, corticosteroids can directly enhance hepatic gluconeogenesis, leading to hyperglycemia.[22] The results of the current study provide insight into possible safety concerns of corticosteroid treatment in a real-world setting for treatment of COPD patients with comorbid diabetes, where both oral and inhaled corticosteroids are utilized. This highlights the possibility for longer-term adverse effects of both oral and systemic corticosteroids on type 2 diabetes complications in patients with diabetes and COPD. Future work should investigate the dose response of corticosteroids and risk for type 2 diabetes in COPD patients.

Our study has a number of limitations. The identification of patients with COPD in our study was based on ≥2 diagnoses of COPD within the Medicare Claims database. However, differences in severity of COPD were not taken into consideration. This is a limitation of this study and deserves consideration as those with more severe COPD are more likely to receive corticosteroids at heightened doses, and thus may be associated with the outcome of diabetes primary diagnosis hospitalizations. While there is a lack of evidence that supports the idea that severity of COPD is on the causal pathway, there is potential that non-biological causal bias exists. This may be the result of clashing priorities, whereby those who have more severe COPD may have limited capacity to focus on management of their comorbid diabetes and therefore have reduced diabetic control. Additionally, after matching cases and controls, four comorbidities were statistically significant (arrhythmias, renal failure, other neurological disorders, and fluid and electrolyte disorders). While there are statistical different this does not indicate clinical differences between both groups.

The association between type 2 diabetes and COPD has vast implications for the clinical management of patients presenting with these morbidities, alone or in combination. Physicians need to take into account the risks and consequences associated with this interaction that may be potentially significant pathophysiologically. Taking into consideration our current understanding of the link between COPD and type 2 diabetes, pneumologists should actively perform screening for type 2 diabetes as a frequent comorbidity of COPD. As COPD patients can be thought of as being at an increased risk for developing diabetes, therapeutic options should be carefully and critically considered, taking into account the risk-to benefit ratios associated with the indication for steroid use.

It might be beneficial to consider the reduction of type 2 diabetes risk as an objective during the selection of therapeutic approaches for COPD. Furthermore, from the point of view of diabetologists, the influence of hyperglycemia on lung function (eg, lung physiology, inflammatory processes and infection) should be considered in patients with diabetes. Indeed, patients could be routinely screened for lung function as a preliminary measure for determining COPD risk. In conclusion, COPD exacerbations are associated with development of type 2 diabetes. Patients with COPD exacerbations with type 2 diabetes should be under regular review to assure that minimally effective doses of corticocorticoids are used. Future studies should assess treatment of patients with type 2 diabetes and COPD to optimize the balance between the risk of benefits and longer-term adverse effects.

## CONTRIBUTORSHIP STATEMENT

The authors would like to thank the Health Informatics and Outcomes Research Academy at UNC Charlotte for access to the Medicare data set. JSM contributed to study design, data interpretation and manuscript writing. CR contributed to data organization and analysis and manuscript writing. BS contributed to study design, data analysis and interpretation, and manuscript writing. CMB contributed to study design, data analysis and interpretation, and manuscript writing.

## FUNDING STATEMENT

This research received no specific grant from any funding agency in the public, commercial or not-for-profit sectors.

## DATA SHARING

No additional data are available.

## COMPETING INTERESTS STATEMENTS

The authors declare no competing interest to declare.
